# Development of Modular Geminivirus‐Based Vectors for High Cargo Expression and Gene Targeting in Plants

**DOI:** 10.1111/pbi.70320

**Published:** 2025-09-21

**Authors:** Matthew Neubauer, Katie Vollen, Chengsong Zhao, José T. Ascencio‐Ibáñez, Linda Hanley‐Bowdoin, Anna N. Stepanova, Jose M. Alonso

**Affiliations:** ^1^ Department of Plant and Microbial Biology North Carolina State University Raleigh North Carolina USA; ^2^ Department of Molecular and Structural Biochemistry North Carolina State University Raleigh North Carolina USA

**Keywords:** beet curly top virus, geminivirus, gene editing, GoldenBraid, transient expression, viral vectors

## Abstract

Viral vectors can be useful tools for expressing recombinant proteins as well as delivering gene‐editing machinery. Despite their utility, the development and subsequent optimisation of these tools is often a difficult and tedious process. Thus, although considerable work has been done to create useful viral vectors for gene editing and protein expression, there is a lack of understanding of how best to design these vectors for specific applications. For instance, it is often unclear whether the inclusion of heterologous promoter sequences or different viral components will improve cargo expression or replicon accumulation. To address some of these hurdles, we designed a GoldenBraid (GB)‐compatible viral vector system based on the geminivirus—beet curly top virus (BCTV). This system allows for simple, modular cloning of a variety of reporter constructs. Making use of this modular cloning strategy, we compared a variety of alternative viral vector architectures. Interestingly, native BCTV promoters outperformed the constitutive *35S* promoter, while the removal of the BCTV virion‐sense genes promoted reporter expression. Intriguingly, these modifications had no effect on total replicon accumulation. These results show the utility of the new modular BCTV‐based vectors for protein expression and gene targeting applications, as well as uncover design principles that may inform future geminivirus‐based viral vector architectures. We anticipate that the availability of this new modular system will spark the broad adoption of replicon‐based strategies in protein expression and gene editing experiments in plants.

## 1 Introduction

Viral vectors have proven useful in various biotechnology applications, such as virus‐induced gene silencing, genome editing and protein expression (Zaidi and Mansoor 2017; Dommes et al. 2019; Abrahamian et al. 2020). Geminiviruses are a family of pathogenic viruses with small circular, single‐stranded DNA genomes that collectively have a wide host range and agricultural impact (Zerbini et al. 2017). Geminivirus‐based viral vectors have been used for the delivery of genome editing sequence‐specific nucleases, such as zinc finger nucleases (ZFNs) and Cas9, as well as repair templates (RT) for homology‐directed repair (HDR) (Butler et al. 2016; Wang et al. 2017; Yu et al. 2020; Gil‐Humanes et al. 2017; Dahan‐Meir et al. 2018; Eini et al. 2022). Furthermore, the ability of geminivirus‐based viral vectors to accumulate at high levels in plant cells has been shown to be critical for providing the high levels of RT necessary for successful gene targeting (GT) in plants (Baltes et al. 2014).

**FIGURE 1:**
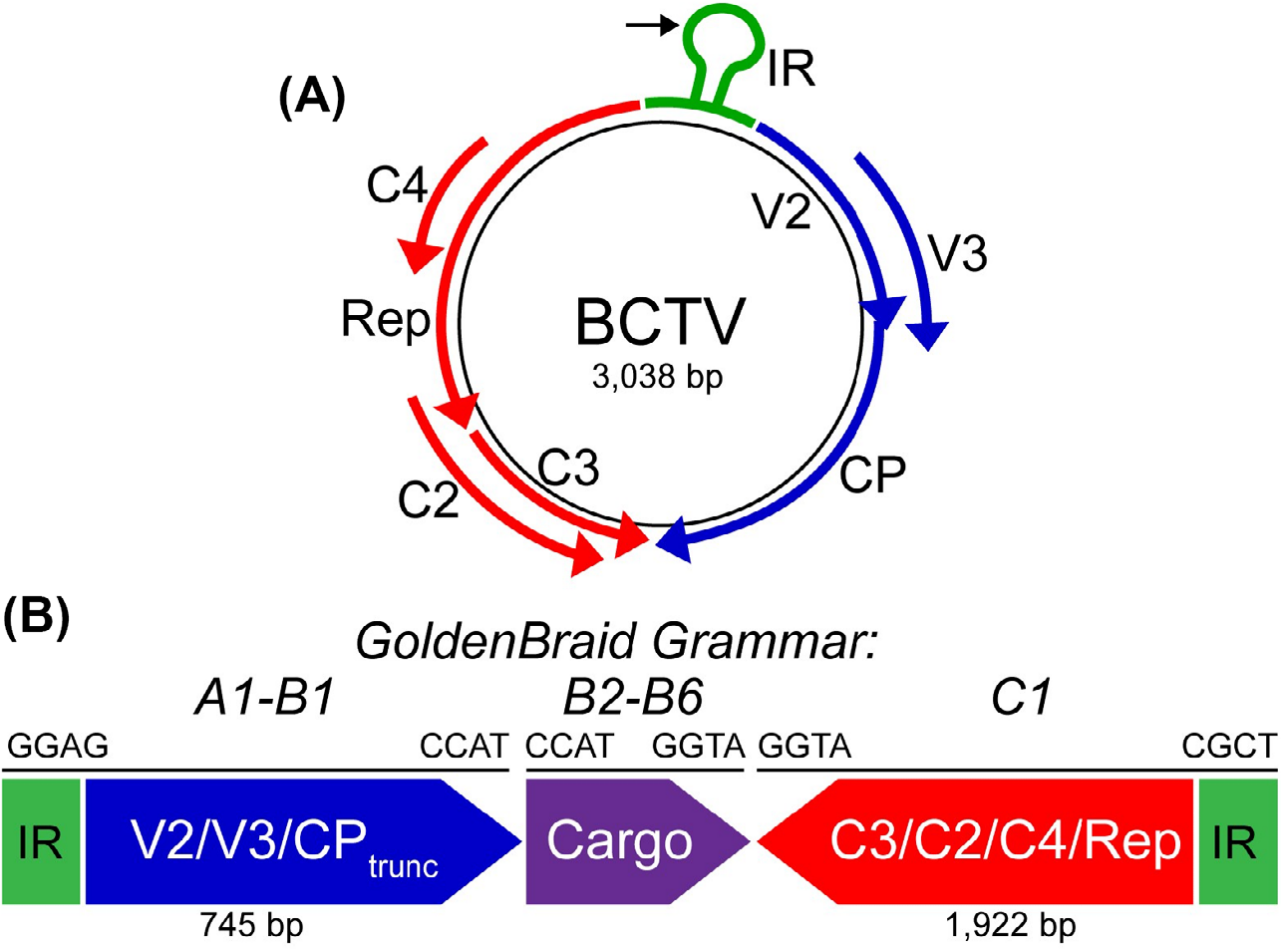
(A) Diagram of the BCTV genome, which is composed of virion‐sense (blue), complementary‐sense (red), genes and an intergenic region (IR, green). The black arrow denotes the location of the nick site near the nonanucleotide sequence that is required for rolling circle replication. (B) GoldenBraid cloning was implemented to generate genetic elements containing portions of the BCTV genome. The virion‐sense (blue) and complementary‐sense (red) genes were cloned with A1‐B1 and C1 grammar, respectively, while the cargo (purple) contained B2‐B6 grammar. The 4‐nt overhangs at the 5′ and 3′ ends of each genetic element are illustrated.

Beet curly top virus (BCTV), a member of the geminivirus family, is a curtovirus with a broad host range and a monopartite genome that consists of virion‐sense and complementary‐sense open reading frames (ORFs), and an intergenic region (IR) (Figure 1A) (Hanley‐Bowdoin et al. 2013). Like other geminiviruses, BCTV replicates by rolling‐circle replication (Hanley‐Bowdoin et al. 2013). The complementary‐sense ORFs encode the Rep (encoded by the *C1* gene), C2, REn (encoded by the *C3* gene) and C4 proteins. The complementary‐sense ORFs are separated from the virion‐sense ORFs by the short AT‐rich IR that contains the origin of replication and viral promoters for bidirectional transcription. The virion‐sense ORFs encode the coat protein (CP, encoded by the *V1* gene), a movement protein (MP, encoded by the *V2* gene) and V3 (Hormuzdi and Bisaro 1993). The virion‐sense ORFs are located directly downstream of the IR, which contains conserved late promoter elements (CLEs) that can be activated following infection (Hur et al. 2008). Of all the DNA elements in the geminivirus genome, only Rep and IR are absolutely required for replication (Gutierrez 1999; Hanley‐Bowdoin et al. 2004), while REn affects the efficiency of this process (Settlage et al. 2005). For most biotechnological applications, efficient replication of the geminivirus‐derived vectors is all that is required. In these cases, the ability to move between cells and support different aspects of the virus life cycle conferred by the other viral genes is not required or desirable. In fact, geminivirus‐derived vectors are often delivered to the plant cell as part of a T‐DNA using *Agrobacterium*‐mediated DNA transfer. Once inside the plant cell, the expression of the T‐DNA‐encoded Rep triggers the rolling‐circle replication of the circularised IR‐flanked sequences corresponding to the engineered geminivirus replicon (Gutierrez 1999; Stenger et al. 1991). Although the genome size of fully functional viruses is constrained by movement requirements (Gilbertson et al. 2003), these constraints are likely removed when the cell‐to‐cell movement and encapsidation of the replicon are no longer of interest. However, the size limits of stable and highly replicative geminivirus‐derived vectors are not known.

Various design approaches have been used to generate BCTV‐based vectors, including removing the virion‐sense genes and inserting heterologous promoters (Kim et al. 2007; Golenberg et al. 2009; Chung et al. 2011; Eini et al. 2022). For instance, a BCTV‐based vector was utilised for Cas12a‐mediated gene editing in plants (Eini et al. 2022). To facilitate the expression of *Cas12a* and other cargoes, the authors removed the virion‐sense genes and inserted a cauliflower mosaic virus (CaMV) *35S* promoter upstream of the cargo (Eini et al. 2022). Similarly, various heterologous promoters were tested for compatibility with BCTV‐based vectors, including CaMV *35S*, the cassava vein mosaic virus promoter and the Arabidopsis *UBIQUITIN3* and *ACTIN8* promoters, with a *GFP* reporter employed to monitor cargo expression levels (Kim et al. 2007). In another study, the authors sought to induce gene silencing by inserting the gene of interest (GOI) in the antisense orientation downstream of a truncated form of the virion‐sense ORFs (Golenberg et al. 2009).

The BCTV IR contains three 30‐bp conserved late element (*CLE*) motifs, which act to drive expression of the virion‐sense genes. These elements alone were shown to be capable of driving the expression of a *GUS* reporter gene in transgenic Arabidopsis lines (Hur et al. 2008). Expression of the reporter was particularly strong in actively dividing tissues and in seedlings. The transcriptional activity of the *CLE* elements was found to diminish as the plant aged. However, infection of mature plants with BCTV restored reporter gene expression. This demonstrates that although the *CLE* elements can mediate high levels of gene expression, this activity is also greatly impacted by the presence of other BCTV components either directly or indirectly through the changes induced in the host cell by the virus (Hur et al. 2008).

GoldenBraid (GB) cloning is based on the use of Type IIS restriction enzymes and modular DNA parts that contain standardised ‘gramma’ of 4‐nt overhangs (Sarrion‐Perdigones et al. 2011, 2013). A major benefit of GB cloning is that it enables existing genetic elements, such as promoters, terminators and tags, to be assembled in the desired order in a single tube reaction and with great efficiency. This allows for previously generated parts to be reused in new constructs without the need to subclone them from scratch. Furthermore, the standardised grammar allows for parts to be easily shared between labs.

In this study, we sought to determine the optimal composition of a BCTV‐based vector for the delivery of repair templates in gene targeting experiments and to enable high levels of expression of a GOI. Utilising the GB cloning approach, we generated and tested a variety of viral vector designs. The inclusion of a heterologous promoter upstream of a GOI was found to be unnecessary, with the intrinsic IR sequence acting as a stronger promoter than the constitutive CaMV *35S*. The BCTV vector was able to accommodate cargo that is 4 kb in size without a reduction in replicon accumulation. Successful GT events were detected following the delivery of gene‐editing machinery by the BCTV‐based vector. Removal of the BCTV virion‐sense genes greatly increased cargo protein expression but did not enhance GT efficiency. These observations demonstrate the utility of tailoring viral vector design depending on its intended use. Furthermore, the broad host range of the BCTV virus compared with other geminivirus (Hanley‐Bowdoin et al. 2013), the modularity of the system and the adoption of the standardised GB technology should facilitate the flexible design and testing of more efficient and application‐customised viral vectors.

## 2 Materials and Methods

### 2.1 Strains, Vectors and Seed Lines

GB cloning vectors, including the pUPD2 entry vector and pDGB3alpha and pDGB3omega vectors, were obtained from the Orzaez lab (Sarrion‐Perdigones et al. 2011, 2013). Additionally, the CaMV *35S* promoter used in this study was derived from the GB0030 clone from the GoldenBraid library. The BCTV sequences used in this study were amplified from a BCTV‐Logan infectious clone (provided by the Hanley‐Bowdoin lab, originally from the D. Bisaro Lab), and cloned into the pUPD2 entry vector. The *Nicotiana tabacum GUS‐NPTII* transgenic line (Wright et al. 2005) and pLSLZDR plasmid were gifts from Dr. Dan Voytas. Wild‐type *N. benthamiana* was grown from the lab stock that originated from seeds provided by Dr. Devarshi Selote.

### 2.2 Plant Growth Conditions

*N. tabacum* and *N. benthamiana* seeds were surface‐sterilised in a solution containing 10% bleach and 0.05% Tween for 1 h, washed three times with sterile dH_2_O, plated on 0.5× MS plates and grown under a 12‐h light cycle. One‐week‐old seedlings were transferred to soil (Sungro Metromix 830) in 24‐cell flats, one seedling per cell and grown at 23°C with 60% humidity under a 16‐h light and 8‐h dark cycle. Four‐week‐old plants were used in transient expression experiments.

### 2.3 Plasmid Construction

GB cloning was employed to generate clones used in this study (Sarrion‐Perdigones et al. 2011). BCTV sequences were subcloned using a BCTV‐Logan infectious clone as a template. The full‐length virion‐sense portion of the genome was amplified using the primers BCTV IR A1F and BCTV 5′ + Stop B2R, incorporating a stop codon (TAA) after the 30th amino acid codon of *CP* (Table S1). To construct virion‐sense deletion (*ΔVS*) clones, the left border‐flanking IR region was amplified using the IRTrunc A1F and IRTrunc B1 R primers (Table S1). Amplification of the complementary‐sense portion of the genome was performed using the BCTV 3′ C1F and BCTV 3′ C1R primers (Table S1). Amplification of the complementary‐sense portion of the genome lacking the IR region was performed using the BCTV 3′ C1F and BCTV C4 C1R primers (Table S1). PCR products were purified and assembled individually into the pUPD2 entry vector.

For later assembly as BCTV cargo, *YPET*, *3xYPET*, *35S* promoter, GT RT and *RUBY* sequences were domesticated with compatible 5′ and 3′ grammar sequences. *YPET* was amplified from an existing pDGB3alpha clone using the YPET B2F and YPET B6R primers or YPET B3F and YPET B6R primers for placement downstream of the *35S* promoter (Table S1). *3xYPET* was amplified from an existing lab pDGB3alpha clone using the 3xYPET B2F and 3xYPET B6R primers (Table S1). The *35S* promoter sequence was amplified from an existing plasmid (GB0030; Addgene plasmid #68163) using the 35sPro B2F and 35sPro B2R primers (Table S1). For use as a GT RT, the RT portion of the pLSLZDR plasmid was amplified using the newRT0 B2F and newRT0 B6R primers (Baltes et al. 2014; Table S1). *RUBY* was cloned as two fragments using the RUBY B2F and RUBY B3R primer pair and the RUBY B4F and RUBY B6R primer pair (Table S1). The template used to amplify the *RUBY* reporter was the *35S:RUBY* plasmid from the Yunde Zhao Lab (He et al. 2020). The ZFN from the pLSLZDR plasmid was amplified using the ZF F B2F and ZF R B5R primers (Baltes et al. 2014; Table S1). Following amplification, these amplicons were assembled into the pUPD2 vector.

PCR‐based site‐directed mutagenesis was performed on pUPD2 entry clones to generate the BCTV virion‐sense ORF and *ΔREP* complementary‐sense clones (Qi and Scholthof 2008). The BCTV Mut F and BCTV Mut R primers were used for the mutagenesis of a BsaI site within the BCTV virion‐sense ORF. To generate *ΔREP* clones, two stop codons were introduced into a pUPD2 clone containing the complementary‐sense ORF with the REP 2STOP F and REP 2STOP R primers (Table S1). Sequencing of BCTV pUPD2 clones was performed using the BCTV Seq 1F, BCTV Seq 1R, BCTV Seq 2F, BCTV Seq 2R and BCTV Seq 3R primers (Table S1). These pUPD2 clones were used in subsequent assemblies to generate pDGB3alpha clones.

Assembly reactions to generate pUPD2 entry clones and pDGB3omega clones were performed using the BsmBI‐v2 Type IIS restriction enzyme and T4 Ligase, New England Biolabs (NEB). Assembly of pDGB3alpha clones was performed using BsaI‐HF and T4 Ligase (NEB). Assembly reactions were performed using a thermocycler set to cycle between digestion and ligation reactions (40 cycles of 37°C for BsaI‐HF or 42C for BsmBI‐v2 for 5 min, followed by 16°C for 5 min).

Assembly of the pDGB3alpha clones *BCTV:YPET*, *BCTVΔREP:YPET*, *BCTVΔIR:YPET*, *BCTVΔVS:YPET* and *BCTVΔVSΔREP:YPET* constructs was performed by assembling viral components previously subcloned into pUPD2 as A1‐B1 and C1 parts, with a pUPD2 clone containing *YPET* cloned as a B2‐B6 part. Assembly of *BCTV:35S:YPET* and *BCTVΔVS:35S:YPET* was performed using pUPD2 clones containing the *35S* promoter and *YPET* cloned as B2 and B3‐B6 parts respectively. The *35S:YPET* pDGB3alpha clone was created by assembling pUPD2 clones containing the *35S* promoter cloned as an A1‐B1 part (GB0030; Addgene plasmid #68163), *YPET* cloned as a B2‐B5 part amplified using the YPET B2F and YPET B5R primers (Table S1) and the *35S* terminator cloned as a B6‐C1 part.

Assembly of the *BCTV:3xYPET* pDGB3alpha clone was performed by combining pUPD2 clones containing the BCTV components cloned as A1‐B1 and C1 parts and the *3xYPET* reporter cloned as a B2‐B6 part. Similarly, the *BCTV:RUBY* and *BCTV:ΔVS:RUBY* pDBG3alpha clones were generated using pUPD2 clones containing the *RUBY* reporter cloned as B2‐B3 and B4‐B6 parts.

To generate clones used for gene targeting experiments, the pDGB3alpha clone *35S:ZF* was assembled using pUPD2 clones containing the *35S* promoter, the ZFN and the *35S* terminator cloned as A1‐B1, B2‐B5 and B6‐C1 parts respectively. The pDGB3alpha clones *BCTV:RT*, BCTV*ΔREP:RT, BCTVΔVS:RT* and *BCTVΔVSΔREP:RT* were assembled using pUPD2 clones containing the viral components cloned as A1‐B1 and C1 parts, with RT cloned as a B2‐B6 part. The pDGB3omega clones *ZFN + BCTV:RT* and *ZFN + BCTVΔVS:RT* were generated by assembling the corresponding alpha clones together.

The DNA sequences of clones used in this study are available online https://benchling.com/mattneub/f_/Gnbpe9gG‐bctv‐entry‐and‐expression‐clones/ (see also Table S2).

### 2.4 Agroinfiltrations and Transient Expression

Plasmids were transformed into *Agrobacterium tumefaciens* strain GV3101 (pMP90) by electroporation with selection on Luria‐Bertani (LB) plates containing 50 μg·mL^−1^ kanamycin sulphate, 20 μg·mL^−1^ gentamicin and 10 μg·mL^−1^ rifampicin. For agroinfiltration experiments, 5‐mL liquid LB cultures were grown for 20 h at 30°C. *Agrobacterium* cells were resuspended in agroinfiltration buffer (10 mM MES, 150 μM acetosyringone, 10 mM MgCl_2_, pH 5.7) at OD_600_ 0.2. After 3 h, *Agrobacterium* was infiltrated into leaves using a needleless 1‐mL syringe. For experiments involving coinfiltration, strains were resuspended in agroinfiltration buffer at OD_600_ 0.2 and then mixed prior to infiltration. Infiltrated plants were kept in the dark for 16 h following infiltration before being returned to a 12‐h light cycle. For GT experiments, an alternate *Agrobacterium* culturing protocol was followed, according to Baltes et al. (2014). Single *Agrobacterium* colonies were grown in 3‐mL LB + antibiotics for 20 h at 30°C with shaking at 250 rpm. Cultures were subcultured by adding 0.5 mL to 25 mL LB + antibiotics + 10 mM MES + 20 μM acetosyringone. After 16 h, cells were pelleted and resuspended in the aforementioned agroinfiltration buffer at OD_600_ 0.2.

### 2.5 Transgenic *GUS‐NPTII N*. *Benthamiana*

The construct used to generate transgenic *GUS‐NPTII N. benthamiana* plants was built using genomic DNA of pre‐existing *N. tabacum GUS‐NPTII* plants as a PCR template (Wright et al. 2005). The broken *GUS‐NPTII* fusion reporter was amplified from genomic DNA using the GUSREP B2F and GUSREP B5R primers and cloned into pUPD2 with GB B2‐B5 grammar (Table S1). This B2‐B5 part was combined with the *35S* promoter and *35S* terminator in the pDGB3alpha1 vector. The binary vector used for *N. benthamiana* transformation was a pDGB3omega2 clone created by combining a plant kanamycin resistance selectable marker with the *35S:GUS‐NPTII* reporter using BsmBI‐v2 (NEB).

Transgenic plants were generated using *Agrobacterium* transformation and subsequent kanamycin selection (Horsch et al. 1989). Ten transgenic lines were generated; PCR analysis confirmed that they all contained the T‐DNA insertion. Agroinfiltration with the *ZFN + BCTV:RT* construct was performed in the T1 generation to identify the lines that performed the best in GT experiments. All lines tested were found to be functional. However, lines 1 and 13 generally gave the best results and were used in subsequent experiments. Transgenic *N. tabacum* and *N*. *benthamiana* seedlings harbouring the *GUS‐NPTII* reporter were selected on 0.5× MS plates supplemented with kanamycin at a final concentration of 50 μg·mL^−1^.

### 2.6 GUS Staining

GUS staining experiments were performed at 7 days postinfiltration (dpi) using a modified protocol (Baltes et al. 2014). Agroinfiltrated leaves were detached and vacuum infiltrated for 5 min with X‐Gluc solution (10 mM phosphate buffer, 10 mM EDTA, 1 mM ferricyanide, 1 mM ferrocyanide, 0.1% Triton X‐100 and 1 mM X‐Gluc (GoldBio)). Vacuum infiltrated leaves were placed in petri dishes containing X‐Gluc solution and incubated in the dark at room temperature with shaking at 80 rpm for the first 2 h. Infiltrated leaves were then incubated at 37°C for 20 h in the dark. After staining, X‐Gluc solution was removed, and the leaves were rinsed with distilled water. Chlorophyll extraction was performed on stained leaf tissue by soaking leaves in 80% ethanol for up to 1 week. The ethanol solution was replaced at least once per day. Stained leaves were imaged using an Olympus digital camera. Successful GT events were quantified by counting the number of blue spots per leaf.

### 2.7 DNA Extraction

To extract DNA, leaf samples were ground in CTAB buffer (1.4 M NaCl, 20 mM EDTA, pH 8, 100 mM Tris–HCl, pH 8, 3% CTAB (cetyltrimethylammonium bromide, Calbiochem)). Tissue grinding was performed by shaking microfuge tubes containing leaf samples and 100 μL of 1‐mm glass beads in a Vivadent dental shaker for 5 s. DNA was isolated from infiltrated leaves through chloroform extraction and DNA precipitation, followed by washing with 70% ethanol as described (Doyle and Doyle 1987).

### 2.8 Quantitative PCR

Measurement of replicon accumulation was performed using qPCR on total DNA. The endogenous *NbFbox* gene was used as a reference gene and was amplified using the primers NbFbox‐F and NbFbox‐R (Eini et al. 2022; Table S1). To measure BCTV replicon accumulation, the LowBCTV‐qRT and UpBCTV‐qRT primers were used (Luna et al. 2017; Table S1). For each experiment, three biological replicates were collected from unique leaves, and three technical replicates were performed on each biological replicate. Reactions were performed using a SYBR Green Master Mix (Applied Biosystems) with a Step One Plus Real‐Time PCR System (Applied Biosystems). qPCR analysis was performed using the Pfaffl method to adjust for discrepancies in primer efficiency (Pfaffl 2001).

### 2.9 Fluorescence Microscopy

Whole‐leaf fluorescence microscopy was performed using a Leica M205 microscope. Images were merged using Leica software. Fluorescence microscopy on smaller leaf sections was performed using a Zeiss AxioImager M.2 upright microscope. ImageJ was employed to quantify fluorescence intensity. For quantification of fluorescence intensity, three equal‐sized regions within the area of infiltration of each construct were quantified. To correct for autofluorescence, three ‘blank’ measurements were taken from un‐infiltrated leaf regions, and the average of these measurements was calculated and subtracted from each of the three fluorescence measurements for each construct. The average of these three corrected fluorescence measurements was then calculated.

### 2.10 DNA Gel Blotting

DNA gel blotting was performed on DNA extracted from *N. benthamiana* leaves that were infiltrated with *Agrobacterium*. DNA was digested with SalI restriction enzyme (NEB) overnight. Gel electrophoresis was performed on digested DNA using a 1% agarose gel. A dry transfer to a positively charged nylon membrane was performed overnight. A PCR DIG probe synthesis kit (Roche) was used for visualisation of the dsDNA probe. The DNA probe was generated by amplifying the target sequence from *N. benthamiana* tissue infiltrated with *Agrobacterium* carrying the *BCTV:YPET* construct using primers BCTV MutR and UpBCTV‐qRT (Table S1).

## 3 Results

### 3.1 Designing a Modular BCTV‐Based Vector

We sought to develop a modular BCTV‐based vector that would enable rapid testing of different cargos and allow targeted alterations to the BCTV machinery encoded within the viral vector. To do this, we utilised a GB‐based approach in which the BCTV virion‐sense genes were cloned as an A1‐B1 part, the cargo was cloned as a B2‐B6 part, and the complementary‐sense genes were cloned as a C1 part to generate the GB BCTV vector (Figure 1B). This modular approach simplifies the implementation of different BCTV‐based vector designs. For instance, many alternative cargoes can be efficiently tested while utilising the same virion‐sense and complementary‐sense fragments. Additionally, modifications can easily be introduced to the viral genetic machinery to optimise the system. Since the GB cloning strategy makes use of both BsaI and BsmBI Type IIS restriction enzymes, the DNA cargos and BCTV sequences that are used must lack these restriction sites. This necessitated the elimination of a single BsaI restriction site present in a virion‐sense ORF. A 2‐nt substitution (TC > AG) was introduced via site‐directed mutagenesis using primers BCTV Mut F and BCTV Mut R (Table S1) to modify this sequence, resulting in a Leucine to Valine substitution at V3 amino acid 42, and leaving V2 unaffected.

### 3.2 Testing New Modular BCTV‐Based Vectors

**FIGURE 2:**
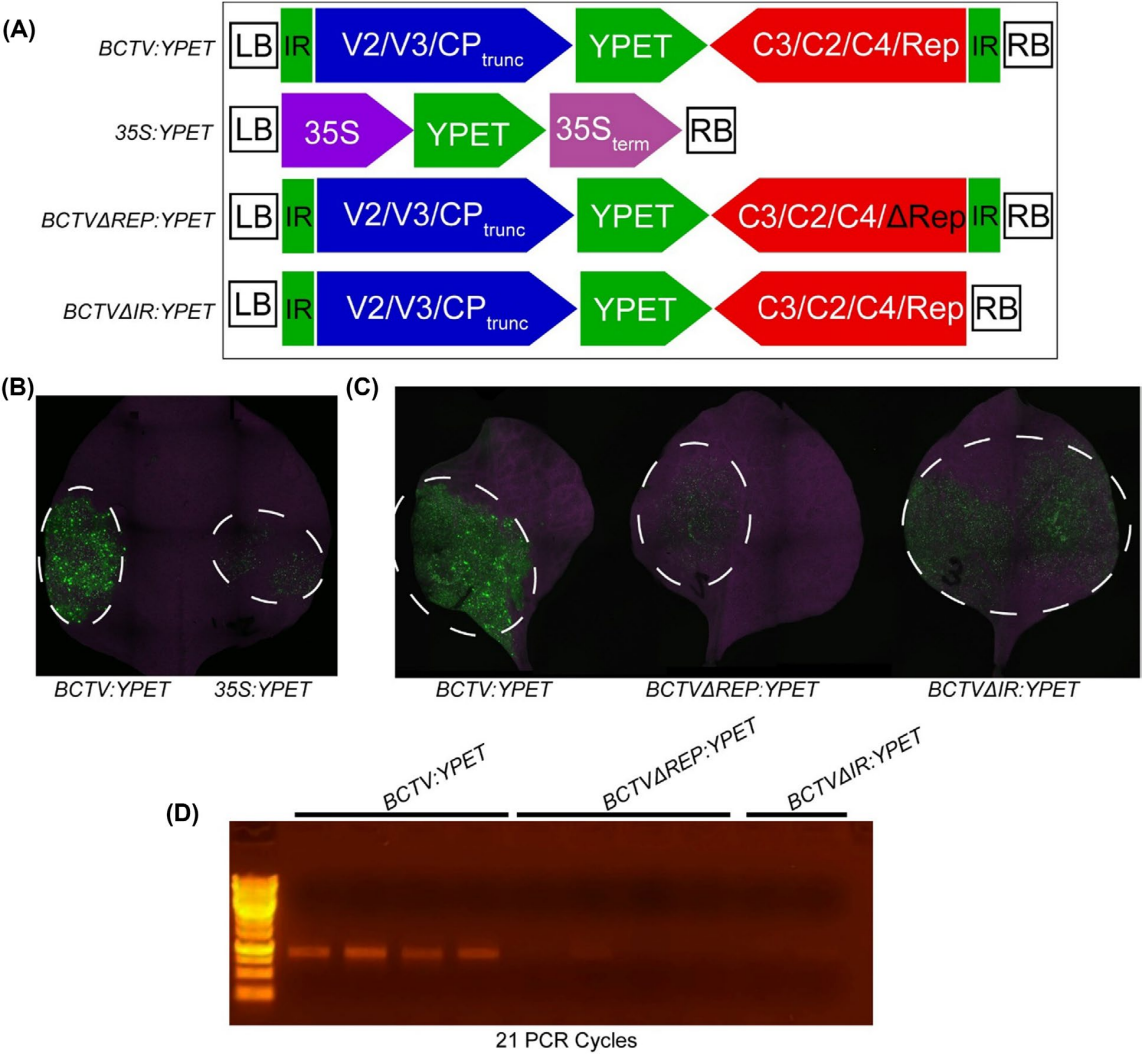
The BCTV vector mediates high levels of reporter expression which is dependent on the accumulation of the replicon. (A) Diagram of the constructs used. A *35S*‐driven *YPET* reporter was used as a control. (B) YPET reporter expression from a BCTV‐based viral vector was stronger than that resulting from a traditional *35S* promoter. Images were taken 5 dpi. (C) Loss of *Rep* and the deletion of the right border‐flanking downstream IR reduce reporter expression. (D) PCR demonstrates that loss of *Rep* and the right‐flanking IR motif inhibit replicon accumulation. PCR was performed using divergent primers that can only amplify the circularised replicon. Each lane represents a unique biological replicate. DNA was extracted from plants at 5 dpi. LB and RB indicate the left and right borders of the T‐DNA. The IR delimits the replicon sequences.

To test whether the modifications made to convert the original BCTV sequences into a GB modular vector affect the basic replicative functions of the virus, we generated a BCTV vector containing a *YPET* reporter gene, *BCTV:YPET*. This construct contains the full‐length BCTV genome; a stop codon (TAA) was inserted into the CP‐encoding gene *V1* after the 30th amino acid codon during PCR amplification to prevent the virus particles from forming (Figure 2A). The *YPET* reporter was inserted, without any promoter or terminator, immediately downstream of the truncated *CP* gene, relying entirely on BCTV's native virion‐sense promoter elements within the IR for its expression. We anticipated that *YPET* expression would be driven by the BCTV promoter normally controlling the transcription of virion‐sense CDSs (*V2/V3/CP*) as part of the polycistron. When tested side‐by‐side with a traditional *35S* promoter‐driven *YPET* T‐DNA construct (Figure 2A) in transient assays in *N. benthamiana*, the *BCTV:YPET* construct consistently gave higher levels of fluorescence (Figure 2B, Figure S2A). We repeatedly observed that the fluorescence in leaves expressing *BCTV:YPET* typically peaked roughly 3–4 days postinfiltration (dpi) compared with a peak at 2 days generated by a *35S:YPET* control construct delivered as a T‐DNA (data not shown). This delay in expression suggested that replicon accumulation is required for the high levels of expression observed in the *BCTV:YPET* construct.

To demonstrate that the enhanced fluorescence observed following *BCTV:YPET* inoculation was due to viral replicon accumulation and not just T‐DNA‐based expression, we generated two replication‐deficient clones: *BCTVΔREP:YPET* and *BCTVΔIR:YPET* (Figure 2A). *BCTVΔREP:YPET* is identical to *BCTV:YPET*, except for two stop codons that were inserted into the 5′ end of the Rep‐encoding *C1* gene, mutating amino acids Cysteine 20 and Glutamine 21 to stop codons (CAGTGT→TAGTGA). As Rep is known to be essential for the initiation of viral replication, its inactivation is expected to abolish rolling circle replication and thus replicon accumulation. *BCTVΔIR:YPET* lacks the IR flanking the T‐DNA right border, which should also greatly inhibit the formation of the replicon. If the fluorescence observed with *BCTV:YPET* is replicon copy number‐dependent, then these clones should exhibit significantly reduced fluorescence. Indeed, fluorescence mediated by the two replication‐deficient clones was dramatically reduced relative to the *BCTV:YPET* clone (Figure 2C, Figure S2B). To verify that these sequence modifications indeed inhibit the formation of the BCTV replicon, PCR was performed on *N. benthamiana* tissue agroinfiltrated with these clones (Figure 2D). The divergent PCR primers used to detect the replicon can produce a product using only the circularised form of the replicon and not the *Agrobacterium*‐delivered binary vector (Figure S1). The lack of efficient PCR amplification suggests that the removal of the functional *Rep‐*encoding *C1* gene or of the right border‐flanking downstream IR significantly inhibits replicon accumulation, and that this reduction likely contributes to the reduction in observed fluorescence (Figure 2C). Taken together, these results demonstrate that the modular, GB‐compatible cloning approach is successful in producing functional BCTV‐based vectors capable of carrying and expressing a promoter‐less cargo sequence and replicating *in planta*.

### 3.3 Testing Alternative Vector Designs

Despite the advantages of viral vectors as tools for plant biotechnology, little is known about how to best design these types of expression cassettes to maximise protein production. For example, in geminivirus expression vectors, the CaMV *35S* or other strong heterologous promoters are often used to drive the expression of the CDS of interest, despite the fact that many viral vectors may include native promoter elements known to drive high production of viral proteins. For instance, the BCTV *IR* is known to contain elements that mediate strong expression of virion‐sense genes (Hur et al. 2007, 2008) that, although not required for replicon formation and rolling circle replication, are involved in many processes, including movement of the virus and the regulation of the ratio of single‐stranded (ss) to double‐stranded (ds) viral DNA (Hormuzdi and Bisaro 1993). Furthermore, the *V2*‐encoded MP protein inhibits host posttranscriptional gene silencing (Hanley‐Bowdoin et al. 2013; Luna et al. 2020). Therefore, although some previously published BCTV‐based vectors lack the virion‐sense genes sequences (Eini et al. 2022), it is possible that including these elements in the construct design will improve replicon formation or cargo expression.

**FIGURE 3:**
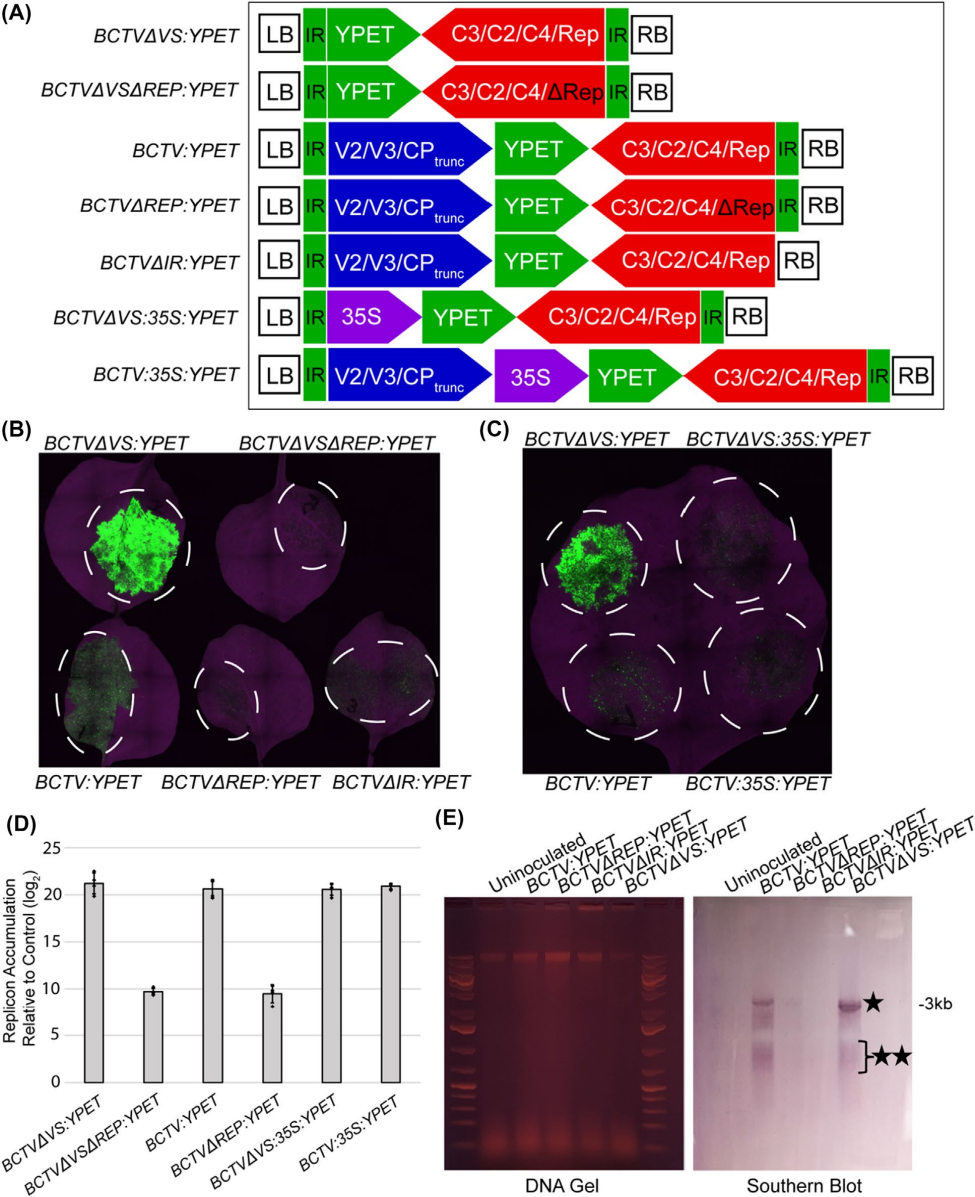
Removal of the BCTV virion‐sense genes improves reporter expression, while inclusion of a heterologous promoter reduces expression. (A) Diagram of various constructs used, including architectures that lack the virion‐sense genes, *Rep* and/or the right‐flanking IR. (B) Transient expression experiments were performed in *N. benthamiana* to determine how various viral vector architectures impact cargo expression. Images were taken at 5 dpi. The strongest expression was observed in leaves infiltrated with the *BCTVΔVS:YPET* construct, followed by the *BCTV:YPET* construct. (C) Inclusion of the *35S* promoter upstream of the reporter reduces expression. YPET fluorescence resulting from constructs containing *35S* promoters was compared with that of constructs without heterologous promoters. BCTV vectors containing the *35S* promoter exhibited reduced fluorescence compared to those without. (D) Insertion of the *35S* promoter upstream of the *YPET* reporter does not result in reduced replicon accumulation. qPCR was performed on DNA extracted from *N. benthamiana* leaves expressing various BCTV vector constructs. Amplification of DNA from constructs lacking *Rep* was significantly reduced, whereas constructs containing the *35S* promoter exhibited amplification levels similar to those without *35S*. Diamonds represent qPCR measurements from individual biological replicates. (E) A DNA gel blot was performed to detect BCTV replicon accumulation in infiltrated *N. benthamiana* leaves. The linearised ~3‐kb dsDNA replicon was detected in leaves infiltrated with functional BCTV constructs (indicated by a black star). A lower molecular weight smearing pattern, consistent with the ssDNA form of the replicon, was also detected (indicated by two black stars). LB and RB indicate the left and right borders of the T‐DNA. The IR delimits the replicon sequences.

To assess the possible utility of native viral promoters in our modified GB BCTV vectors, we tested various construct architectures, taking advantage of the modular nature of the GB system and the ease with which different domesticated DNA parts can be assembled in the desired order (Figure 3A). In the first design, *BCTVΔVS:YPET*, a promoterless *YPET* CDS was placed between the left border‐flanking IR and the *C3/C2/C4/Rep* sequences as an A1‐B6 GB part, replacing the *V2/V3/CP*_*trunc*_. A version of this construct lacking a functional *Rep* was also included, *BCTVΔVS:ΔREP:YPET*. In these constructs, *YPET* expression would be driven by the BCTV promoter normally controlling the transcription of virion‐sense CDSs (*V2/V3/CP*) (Figure 3A).

To test how removing the virion‐sense CDSs would impact reporter expression, *Agrobacterium*‐mediated transient expression experiments were performed in *N. benthamiana*. Leaves were imaged at 5 dpi. In *N. benthamiana* transient assays, we observed that the *BCTVΔVS:YPET* construct consistently demonstrated higher reporter expression than the *BCTV:YPET* construct retaining the virion‐sense genes (Figure 3B, Figure S2C). The loss of *Rep* in the *BCTVΔVSΔREP:YPET* construct severely reduced the YPET fluorescence (Figure 3B), as was observed previously with the *BCTVΔREP:YPET* and *BCTVΔIR:YPET* vectors (Figure 2C), indicating that the high expression levels require replicon accumulation (Figure 3B, Figure S2C).

Additional architectures were tested to determine whether heterologous promoters would impact cargo expression. In one such design, *BCTV:35S:YPET*, a *35S:YPET* transcriptional unit was used as the GB B2‐B6 part placed downstream of virion‐sense genes (*V2/V3/CP*_*trunc*_) (Figure 3A). In parallel, a similar architecture was constructed, *BCTVΔVS:35S:YPET*, in which a *35S:YPET* transcriptional unit was placed directly downstream of the left border‐flanking IR in place of the virion‐sense genes (Figure 3A). To test whether insertion of a heterologous *35S* promoter could enhance reporter expression, these constructs were evaluated using *Agrobacterium*‐mediated transient assays (Figure 3C). We found that the constructs containing the *35S* promoter performed worse than their counterparts without the *35S* promoter (Figure 3C, Figure S2D). This reduction in reporter expression was particularly evident in constructs lacking the virion‐sense genes, with a ~10 fold decrease in YPET fluorescence (*BCTVΔVS:YPET/BCTVΔVS:35S:YPET*), relative to a ~2 fold reduction for the full‐length viral constructs (*BCTV:YPET/BCTV:35S:YPET*).

These results demonstrated that removal of the virion‐sense genes has a positive impact on reporter expression, while the inclusion of the *35S* promoter upstream of the reporter has a detrimental effect. These changes in reporter expression could be the result of altered replicon accumulation. To test this, qPCR assays were performed on DNA extracted from *N. benthamiana* leaves infiltrated with *Agrobacterium* strains carrying various BCTV‐based vector constructs (Figure 3D). As a control, we used the endogenous *NbFbox* gene as a reference gene for normalisation (Eini et al. 2022). qPCR demonstrated that despite mediating significantly different levels of reporter expression, the *BCTV:YPET*, *BCTVΔVS:YPET*, *BCTV:35S:YPET* and *BCTVΔVS:35S:YPET* constructs supported similar levels of replicon accumulation (Figure 3D). Using the *BCTVΔVSΔREP:YPET* and *BCTVΔREP:YPET* constructs as a baseline, we estimate that the copy number of the replicon was roughly 4000 copies per plant genome. These results indicate that the differences observed in reporter gene expression among these four constructs are not the result of changes to replicon accumulation and could instead be due to some other factors, such as ratios of ss and ds replicon or different transcriptional activity of the promoter architectures tested.

As mentioned above, one explanation for the differences in reporter expression mediated by the various BCTV vectors is differences in the ratio of ds and ss replicons that accumulate within the cell. Although BCTV replicons exist as both ds and ss DNA within the cell, it is the ssDNA form of the replicon that is packaged into the CP (Bennett and Agbandje‐McKenna 2020). Importantly, the host cell machinery expresses the viral genes using the dsDNA replicon as a template (Hanley‐Bowdoin et al. 2013). Genes encoded in the BCTV virion‐sense ORF are known to be involved in regulating the ratio of ss and ds DNA (Stanley and Latham 1992; Hormuzdi and Bisaro 1993). Since the *BCTVΔVS:YPET* construct lacks these genes, it is possible that the replicon preferentially accumulates in the dsDNA form in these deletion constructs, potentially explaining the enhanced YPET fluorescence. To test this possibility, a DNA gel blot was performed on DNA extracted from *N. benthamiana* leaves that were infiltrated with *Agrobacterium* carrying various BCTV‐based vectors. Prior to DNA gel blotting, the DNA extract was treated with SalI restriction enzyme overnight to linearise the dsDNA. Both *BCTV:YPET* and *BCTVΔVS:YPET* contain a single SalI restriction site. The results of the DNA gel blot analysis demonstrated that although the *BCTV:YPET* and *BCTVΔVS:YPET* replicons accumulate at similar levels (Figure 3D), *BCTVΔVS:YPET* preferentially accumulates in a dsDNA form that is visible as a single 3‐kb band (Figure 3E). On the other hand, *BCTV:YPET* produces a fainter 3‐kb band and preferentially exists in the ssDNA form that is visible as a smearing pattern that migrates faster than the 3‐kb band (Stanley and Latham 1992). This difference in the ratio of ssDNA and dsDNA produced by the two vectors is another possible explanation for the difference in reporter gene expression and should be used to inform future vector design.

### 3.4 Determining the Size Limit of the BCTV Replicon

**FIGURE 4:**
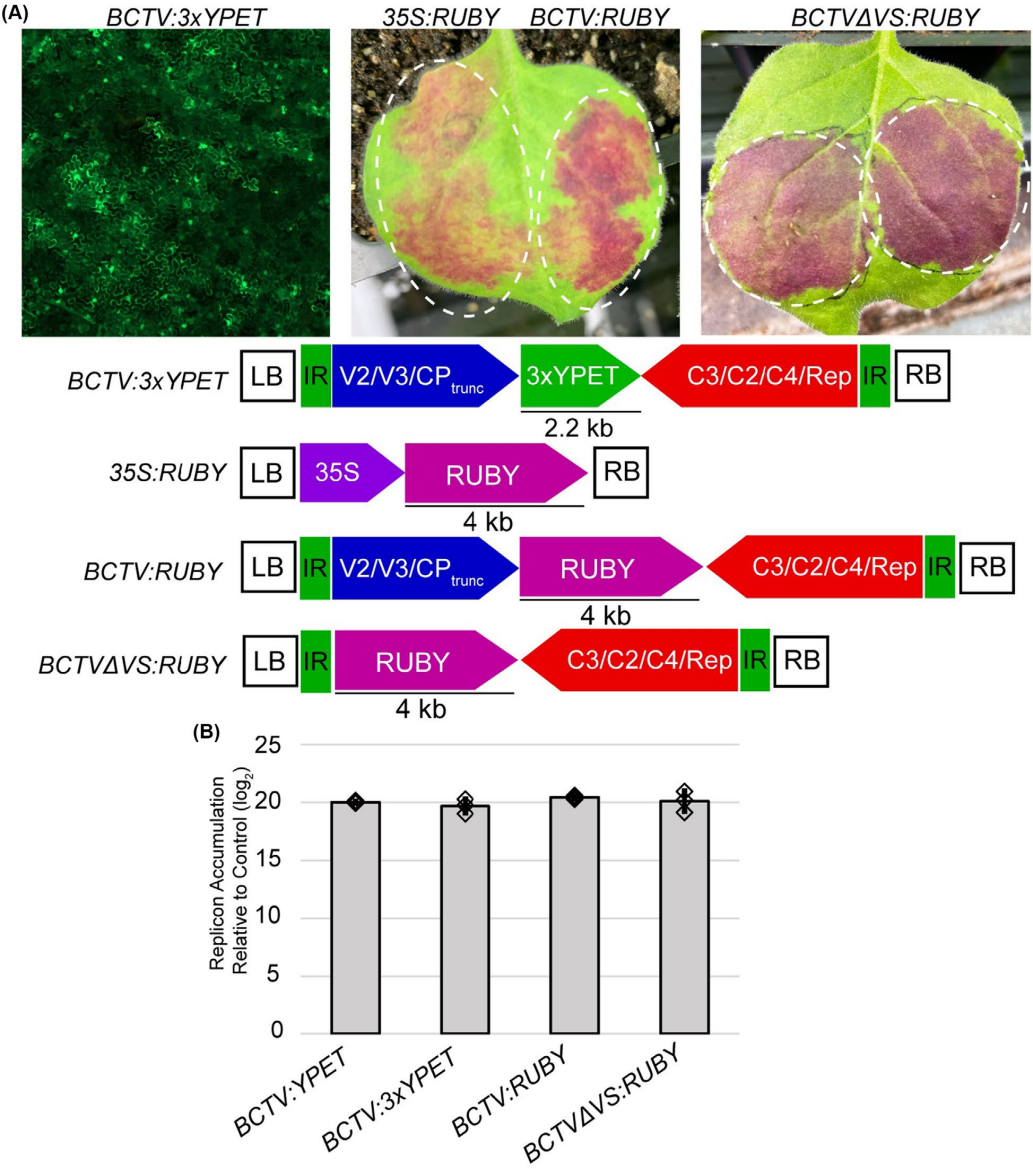
The BCTV vector can accommodate large cargos of at least 4 kb. (A) Fluorescence micrograph from a *N. benthamiana* leaf expressing a BCTV vector containing a triple *YPET* reporter, *BCTV:3xYPET* (left panel). Leaves expressing the *RUBY* reporter under the control of the *35S* promoter (left half of the middle panel), placed downstream of the truncated virion‐sense genes (right half of the middle panel) or directly downstream of the left border‐flanking IR (right panel). White dashed lines denote the infiltrated areas of the leaf. (B) BCTV vectors with various cargoes accumulate to similar levels. qPCR was performed to determine whether the size of the cargo in the viral vector impacts BCTV accumulation. Diamonds represent qPCR measurements from individual biological replicates. DNA was extracted from leaves at 5 dpi. LB and RB indicate the left and right borders of the T‐DNA. The IR delimits the replicon sequences.

A major drawback of using viral vectors is that they can have restrictive cargo size limitations that prevent their use in certain applications. To determine the limits of our BCTV vector as well as to test our modular cloning approach, two BCTV‐based constructs with large reporter genes were constructed: *BCTV:3xYPET* and *BCTV:RUBY*. *BCTV:3xYPET* contains a 2.2 kb triple *YPET* reporter composed of three individual *YPET* CDSs fused together (Zhou et al. 2011). The *BCTV:RUBY* construct contains a 4‐kb *RUBY* reporter (He et al. 2020), a chimeric sequence that encodes three enzymes involved in the production of a reddish‐purple pigment, betalain (Figure 4A). These constructs were evaluated using *Agrobacterium*‐mediated transient expression in *N. benthamiana*.

The *BCTV:3xYPET* and *BCTV:RUBY* constructs successfully mediated reporter expression (Figure 4A). When compared to a *35S:RUBY* construct delivered using a traditional T‐DNA vector, the *BCTV:RUBY* vector resulted in stronger reporter expression. The *BCTVΔVS:RUBY* construct was also able to mediate strong expression of the *RUBY* reporter (Figure 4A). Given the size of these cargoes, it is possible that their inclusion in the viral vector has a negative impact on replicon accumulation. To test whether the inclusion of these large cargos negatively impacts replicon accumulation, qPCR was performed on DNA extracted from agroinfiltrated *N. benthamiana* leaves (Figure 4B). qPCR analysis demonstrated that there was no prominent difference in replicon accumulation between the *BCTV:YPET*, *BCTV:3xYPET, BCTV:RUBY* and *BCTVΔVS:RUBY* vectors. This indicates that a cargo size of up to 4 kb does not have a significant impact on the ability of the replicon to accumulate within the cell. Although it remains unclear what the upper size limit may be, the fact that the BCTV replicon was able to handle the *RUBY* reporter indicates that our modular BCTV vectors can tolerate cargos of at least 4 kb in size, which is consistent with prior observations that cell‐to‐cell movement is the primary limiting factor for geminivirus genome size (Gilbertson et al. 2003).

### 3.5 Use of the BCTV Vector in Gene Editing

Viral vectors, in addition to serving as useful tools for mediating high levels of protein expression, can also be leveraged as delivery vehicles for gene editing machinery. We sought to determine whether our BCTV‐based vector could be used to deliver a RT for GT, as well as whether the inclusion of the BCTV virion‐sense genes has an impact on GT efficiency since these genes can affect the proportion of ss and ds viral DNA that accumulates in the plant cell (Hormuzdi and Bisaro 1993).

**FIGURE 5:**
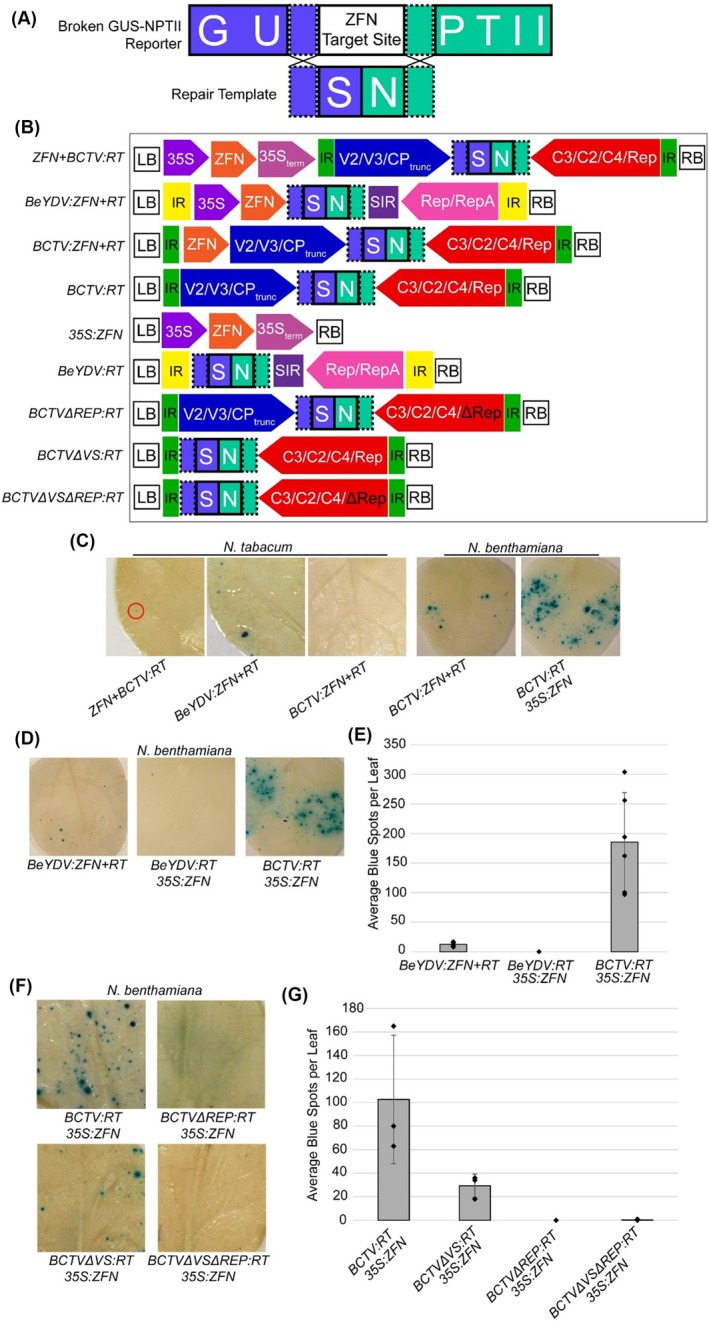
Optimisation of the BCTV vector design for gene targeting (GT). (A) Diagram of the broken *GUS* reporter construct used to detect successful GT events. The *GUS* reporter lacks sequence that is required for GUS activity, but it can be repaired if the repair template (*RT*) is used in the repair of the double‐strand break induced by the ZFN. (B) Diagram of the various BCTV and BeYDV constructs used for GT experiments. (C) BCTV‐delivered RT can successfully mediate GT in *N. tabacum* and *N. benthamiana*. The BCTV‐based construct was less effective than the BeYDV‐based vector in *N. tabacum* . The BCTV‐based vector achieved higher levels of GT when the *ZFN* was removed from the replicon and delivered as a separate *35S:ZFN* construct. Images of representative leaves subjected to GUS‐staining are shown following chlorophyll removal, with a red circle indicating GUS‐stained tissue. (D) BCTV‐based viral vectors mediate higher levels of GT than BeYDV‐based vectors in *N. benthamiana*. Leaves were infiltrated with *Agrobacterium* carrying the corresponding vectors. BeYDV‐based GT was more efficient when the *ZFN* was included within the BeYDV replicon than when a separate vector encoding the ZFN was provided. (E) Quantification of successful GT events resulting from BeYDV and BCTV constructs. Co‐infiltration of *BCTV:RT* and *35S:ZFN* resulted in greater GT efficiency than *BeYDV:ZFN + RT* and co‐infiltration of *BeYDV:RT* with *35S:ZFN*. Moving the *ZFN* outside of the BeYDV replicon reduced the GT efficiency. (F) GT mediated by the BCTV vector requires *Rep* and is enhanced when the virion‐sense genes are included. Leaves were coinfiltrated with a construct containing a *35S*‐driven *ZFN* and the corresponding BCTV construct. Images of representative GUS‐stained leaves are shown following chlorophyll removal. (G) Quantification of successful GT events resulting from various BCTV constructs. Loss of *Rep* greatly reduced GT events. More successful GT events occurred when the BCTV virion‐sense genes were included in the construct. Diamonds represent qPCR measurements from individual biological replicates. LB and RB indicate the left and right borders of the T‐DNA. The IR delimits the replicon sequences. Construct names separated by a ‘+’ indicate that they were part of the same T‐DNA, while the cotransfection with two different T‐DNAs is depicted by placing the name of the constructs in two separate text lines.

To monitor HDR‐based gene editing, we used a reporter line generated previously in *N. tabacum* (Wright et al. 2005). This reporter harbours a ‘broken’ *GUS‐NPTII* fusion that lacks part of the sequence necessary for enzymatic activity (Figure 5A). The reporter also contains a ZFN recognition site that can be cleaved to mediate gene editing. If successful GT occurs, the broken reporter is repaired, the inactive GUS enzyme is made active, and GT events can be detected by GUS staining.

To test the suitability of our BCTV‐derived vector for GT experiments, we generated two constructs. In the first construct, *ZFN + BCTV:RT*, the *ZFN* was placed upstream of the replicon and was driven by the *35S* promoter. In the second construct, *BCTV:ZFN + RT*, both the *35S:ZFN* and the *RT* were included in the replicon, and the *35S:ZFN* was placed directly downstream of the IR (Figure 5B). The *RT* and *ZFN* used in these experiments were derived from the previously generated pLSLZDR vector based on the bean yellow dwarf virus (BeYDV) sequence (Baltes et al. 2014). To test whether these new BCTV‐based vectors can mediate successful GT, they were delivered into the broken *GUS* reporter *N. tabacum* line using *Agrobacterium*‐mediated transient expression. After GUS staining, occasional blue spots were observed in leaves transformed with *ZFN + BCTV:RT* and *BeYDV:ZFN + RT*, indicating that HDR took place (Figure 5C). In leaves expressing the *BCTV:ZFN + RT* construct, there were no visible blue spots, indicating that including the *ZFN* within the BCTV replicon may reduce the GT efficiency. The efficiency of GT using the new BCTV‐based vectors was much lower than that observed when using the previously published BeYDV‐based pLSLZDR vector *BeYDV:ZFN + RT* (Figure 5C). These results suggest that BCTV may not function as effectively as BeYDV in *N. tabacum* , or that neither of the two BCTV‐derived vector architectures used in these experiments was optimal for the delivery of appropriate levels of the GT machinery.

To test whether the low GT efficiency of BCTV‐based vectors could be species‐specific, the ‘broken’ *GUS:NPTII* reporter from *N. tabacum* was subcloned into pDGB3a1 and transformed into *N. benthamiana*. We selected two independent lines that performed well in GT experiments using the *ZFN + BCTV:RT* construct (Figure S3). Interestingly, GT efficiency in these lines was much higher than that observed in the previously generated *N. tabacum* lines (Figure 5C), suggesting that the GT efficiency of the BCTV‐derived vectors is highly dependent on the plant species. We also decided to further test the effect of the vector architecture on the GT efficiency. We had observed that, although still very low, the GT efficiency in *N. tabacum* when the *ZFN* was placed outside the replicon (i.e., for the *ZFN + BCTV:RT* construct) was higher than when the *ZFN* was located inside (*BCTV:ZFN + RT*) (Figure 5C). Therefore, we decided to test a third vector architecture where the *ZFN* was expressed from a separate T‐DNA. We thus assembled a new BCTV‐based vector, *BCTV:RT*, in which the T‐DNA carrying the replicon no longer includes the *ZFN*. We subsequently coinfiltrated *N. benthamiana* with *Agrobacterium* strains carrying *BCTV:RT* and *35S:ZFN*. To our surprise, co‐infiltration with these two strains resulted in consistently higher GT efficiencies than those obtained with the *ZFN + BCTV:RT* (Figure 5C).

To further examine the effects of the viral vector, its architecture and the plant species on GT efficiency, we generated a new BeYDV‐derived vector by removing the *ZFN*, resulting in the *BeYDV:RT* construct (Figure 5A). Using the transgenic *N. benthamiana* GUS reporter lines, we then examined the GT efficiency of the original *BeYDV:ZFN + RT*, in which both the *ZFN* and the *RT* are located inside the replicon, along with the coinfiltration of either the new *BeYDV:RT* with the *35S:ZFN* or the *BCTV:RT* with the *35S:ZFN*. As we have described above, the co‐infiltration of *BCTV:RT* with *35S:ZFN* resulted in high levels of GT in *N. benthamiana*. Interestingly, these levels of GT were consistently higher than those obtained with the original *BeYDV:ZFN + RT*, which, in turn, were higher than those obtained when co‐infiltrating with *BeYDV:RT* and *35S:ZFN*, with the latter combination failing to produce any GUS‐positive recombination spots (Figure 5D,E). Together, these results further highlight the importance of the host plant species, as well as viral vector origin and architecture, that is, factors that are likely determinants of the expression levels of the various components required for GT.

Given that the removal of the BCTV virion‐sense genes has a positive impact on cargo expression (Figure 3B), we questioned whether it may also have a positive impact on GT efficiency. To test this, we co‐infiltrated leaves with *Agrobacterium* strains carrying either *BCTVΔVS:RT* and *35S:ZFN* or *BCTV:RT* and *35S:ZFN*. We observed that the *BCTV:RT* construct consistently mediated greater GT efficiency than the *BCTVΔVS:RT* construct lacking the virion‐sense genes (Figure 5E,F). This suggests that although the loss of the BCTV virion‐sense genes greatly enhances protein expression, it has the opposite effect on GT efficiency.

Having demonstrated that BCTV‐based vectors can mediate high levels of GT in *N. benthamiana*, we then tested whether this GT efficiency was directly related to the ability of the replicon to accumulate at high levels within the cell (Figure 5F,G). To test this, we generated two additional constructs that lack a functional *Rep* gene due to two in‐frame stop codons in the 5′ end of the gene, *BCTVΔREP:RT* and *BCTVΔVSΔREP:RT*. As expected, loss of *Rep* prevented successful gene targeting events in *N. benthamiana* (Figure 5F,G), demonstrating that successful GT is dependent upon the copy number of the replicon‐provided repair template.

Taken together, these results demonstrate that BCTV‐derived vectors can be used for efficient GT; and that BCTV and BeYDV GT efficiency is highly dependent on the plant species and viral vector architecture.

## 4 Discussion

Viral vectors have the potential to improve various fields of plant biotechnology, including gene editing and molecular farming. Successful gene targeting requires that a repair template and gene editing machinery be delivered to the cell, both of which can be mediated by viral vectors. Another promising use for viral vectors is in promoting high levels of protein expression. The use of plants to express important proteins is a potential way to produce pharmaceuticals and other valuable compounds (Abrahamian et al. 2020). A greater understanding of how to design viral vectors can promote their use in plant biotechnology.

Previously generated viral vectors have generally been constructed using traditional cloning methods that can complicate the insertion of desired cargoes or alterations of the viral sequences. Here, we utilised a GB cloning approach to simplify the construct generation process. GB cloning has previously been used to create BeYDV‐based vectors (Dusek et al. 2020). This approach enabled the authors to test different cargoes, but no alternative viral components were evaluated in that study. Since previous BCTV vector designs have included heterologous promoter sequences upstream of the cargo (Kim et al. 2007; Eini et al. 2022), we were interested in examining whether cargo expression is enhanced by these modifications. Interestingly, when we tested the inclusion of the widely used *35S* promoter upstream of the cargo, we found that it reduced cargo expression, despite having no effect on replicon accumulation (Figure 3C). Given that the IR motif contains elements that enable strong expression of the virion‐sense genes, it is likely that the native elements in this motif mediate stronger expression than nonnative elements like the *35S* promoter. In addition to improving cargo expression, the use of the native promoter elements also results in a smaller overall replicon size. Future vector designs, including those based on BCTV and other geminiviruses, should benefit from this observation.

BCTV machinery is involved in many different processes, including the replication of the viral replicon, manipulation of the host and controlling the ratio of ds and ssDNA. We found that changes to the architecture of the vector, including the removal of the BCTV virion‐sense genes, impact cargo expression. Removal of the BCTV virion‐sense ORFs, including *V2* and *V3*, enhanced reporter expression while having no impact on replicon accumulation (Figure 3B,D). This may be due to the cargo being placed closer to the IR motif, which controls the expression of the virion‐sense genes. We also observed that the removal of the BCTV virion‐sense genes impacts the ratio of ds and ssDNA replicon that accumulates (Figure 3E). Thus, another possible explanation for the enhanced cargo expression in the BCTV clones lacking the virion‐sense genes is that they provide more dsDNA to be transcribed by the host machinery.

Although we observed greater protein expression in BCTV constructs lacking the virion‐sense genes, this architecture performed worse in GT experiments. A possible explanation for this is that the ssDNA replicon generated by constructs containing the virion‐sense genes may serve as a more efficient template for GT than a dsDNA replicon. There is evidence to suggest that ssDNA templates may be more efficient for GT than dsDNA templates. In *Chlamydomonas reinhardtii* , Cas12‐mediated GT was boosted 500‐fold using ss oligodeoxynucleotides (ssODNs) (Ferenczi et al. 2017). Efficient GT using ssDNA templates in conjunction with a ZFN has also been demonstrated in human cell lines (Chen et al. 2011).

Another important consideration when using vectors derived from viruses is their cargo size limits. In general, the genome size, and therefore, the DNA cargo that viruses can efficiently pack into infectious particles, is typically limited by the requirements of plasmodesmata and the MP (Gilbertson et al. 2003). On the other hand, although the encapsidation restrictions would be irrelevant in virus‐derived vectors lacking coat protein genes and unable to form infectious particles, other aspects of the virus biology may still impose certain cargo size limitations. For instance, large cargo sizes may affect the replication efficiency or the structural fidelity of the formed replicons. Here, we have shown that relatively large cargos of ~4 kb do not significantly affect replicon accumulation or cargo protein expression, suggesting that most of the replicons formed contain the intact cargo sequence. We have also demonstrated that the deletion of the virion‐sense genes does not have detrimental effects on cargo expression or replicon accumulation, suggesting that cargos even bigger than 4 kb could be accommodated in this BCTV‐derived vector, making this new vector suitable for a large number of applications that require several kilobases of cargo DNA. It is also important to note that the stability, replication and structural integrity of the replicons would likely depend on the specific sequences cloned in this vector. Other factors beyond cargo size might also influence replication efficiency. Therefore, future studies should explore not only the effects of a wide range of DNA sizes but also the GC content, abundance of repetitive DNA, and other DNA features on replicon abundance and sequence stability.

Overall, these observations demonstrate the importance of designing a viral vector with its intended purpose in mind, whether that be to increase protein expression or deliver repair templates for gene targeting. The use of GB cloning enabled us to test numerous replicon architectures efficiently and optimise the BCTV vector for use in various applications. A similar approach could prove beneficial for designing and building more effective viral vectors for other plant viruses, host plants and biotech applications, including the rapidly increasing array of CRISPR‐based tools. Finally, the modularity of the system will also facilitate the modification required for the use of these constructs in stable transformation experiments, such as the inclusion of plant selectable markers, or the precise spatiotemporal control of the virion replication and cargo expression.

## Author Contributions

Experiments were performed by M.N., K.V., C.Z. and J.T.A.‐I. Conceptualisation, experimental design and manuscript preparation were carried out by M.N., K.V., C.Z., J.T.A.‐I., L.H.‐B., A.N.S. and J.M.A.

## Conflicts of Interest

The authors declare no conflicts of interest.

## Supporting information


**Table S1:** Primer table.
**Table S2:** Sequence table.

**Figure S1:** pbi70320‐sup‐0002‐FiguresS1‐S3.pdf.
**Figure S2:** pbi70320‐sup‐0002‐FiguresS1‐S3.pdf.
**Figure S3:** pbi70320‐sup‐0002‐FiguresS1‐S3.pdf.

## Data Availability

The data that support the findings of this study are available on request from the corresponding author. The data are not publicly available due to privacy or ethical restrictions.
