# Supplementary material for: Development of Modular Geminivirus‐Based Vectors for High Cargo Expression and Gene Targeting in Plants
Source: Plant Biotechnol J. 2025 Sep 21;24(2):717–30. doi: 10.1111/pbi.70320 (PMC12906812; doi:10.1111/pbi.70320)
Supplement: Supplementary file 1 — Table S1: Primer table. Table S2: Sequence table. [file PBI-24-717-s002.docx]

**Table S1.** Primer Table

| **Primer Name** | **Sequence (5'-3')** | **Description** |
| --- | --- | --- |
| BCTV Seq 1F | GGGCACCCAATCAAAACGTGAC | For sequencing BCTV components |
| BCTV Seq 1R | CCTCGCCTTCTTCTAGGGTCAG | For sequencing BCTV components |
| BCTV Seq 2F | GCAACACTCTGGTTCTGAATTAC | For sequencing BCTV components |
| BCTV Seq 2R | CCTCATCTCCACGTCCTGCTTC | For sequencing BCTV components |
| BCTV 3' C1F | GCGCCGTCTCGCTCGGGTATAATATTTATTATTAATAATGAAATTAGTACAAGTTC | For subcloning the complementary-sense ORFs |
| BCTV 3' C1R | GCGCCGTCTCGCTCAAGCGATATACATGTAAAAATAACGTATATAAAACG | For subcloning the complementary-sense ORFs |
| BCTV IR A1F | CGGCCGTCTCGCTCGGGAGCCGTTATAATATTACCGGATGG | For subcloning the virion-sense ORFs |
| BCTV 5' +Stop B2R | GCGCCGTCTCGCTCAATGGTTAGCTAGTCCTCCTCTTCTT | For subcloning the virion-sense ORFs; truncates the CP and adds a stop codon |
| BCTV Mut F | GTCAGTGACCCTAGAAGAAGGCG | For mutagenizing BsaI sites within virion-sense ORFs |
| BCTV Mut R | CTGACCCTATCTCTTGATGGATACC | For mutagenizing BsaI sites within virion-sense ORFs |
| BCTV Repl F | GGAGGCATTGGACTCCGATG | For amplification of the BCTV replicon |
| Ruby B2F | GCGCCGTCTCGCTCGCCATGGATCATGCGACCCTCGC | For subcloning *RUBY* |
| RUBY B3R | GCGCCGTCTCGCTCAGGCTGTCTCGAACCCGGACT | For subcloning *RUBY* |
| RUBY B4F | GCGCCGTCTCGCTCGAGCCGAGACAGACCAAAAGA | For subcloning *RUBY* |
| RUBY B6R | GCGCCGTCTCGCTCATACCTCACTGGAGGCTTGGCTCAA | For subcloning *RUBY* |
| RUBY 3300R | CTGAGCCACTGCACGAACTC | For subcloning *RUBY* |
| 3xYPET B2F | GCGCCGTCTCGCTCGCCATGTCTAAGGGTGAAGAGTTG | For subcloning triple *YPET* |
| 3xYPET B6R | GCGCCGTCTCGCTCATACCTTACTTGTAAAGTTCATTCATGCC | For subcloning triple *YPET* |
| newRT0 B2F | GCCGCGTCTCGCTCGCCATGGCGTGCCTGCAGACTAGTA | For subcloning a repair template for the *GUS:NPTII* fusion |
| newRT0 B6R | GCGCCGTCTCGCTCATACCCTCTCCCGGGATTACCCTGT | For subcloning a repair template for the *GUS:NPTII* fusion |
| YPET B6R | GCGCCGTCTCGCTCATACCTCACTTATACAATTCATTCATTCC | For subcloning *YPET* |
| YPET B2F | CGGCCGTCTCGCTCGCCATGTCAAAGGGAGAGGAACTTTTC | For subcloning *YPET* |
| YPET B5R | GCGCCGTCTCGCTCAAAGCTCACTTATACAATTCATTCATTCCTT | For subcloning *YPET* |
| 35sPro B2 F | GCGCCGTCTCGCTCGCCATACTAGAGCCAAGCTGATCTC | For subcloning the *35S* promoter used in the BCTV replicon |
| 35sPro B2 R | GCGGCGTCTCGCTCACATTCAGCGTGTCCTCTCCAAATG | For subcloning the *35S* promoter used in the BCTV replicon |
| YPET B3 F | CGGCCGTCTCGCTCGAATGTCAAAGGGAGAGGAACTT | For subcloning *YPET* used in the *35S:YPET* construct |
| IRTrunc A1 F | GCGCCGTCTCGCTCGGGAGCCGTTATAATATTACCG | For subcloning the left-border-flanking IR region to construct *ΔVS* constructs |
| IRTrunc B1 R | GCGCCGTCTCGCTCAATGGTTATAAGTACATATACATGTAAAAAAAATGTA | For subcloning the left-border-flanking IR region to construct *ΔVS* constructs |
| BCTV Seq 3R | CGGATGGCCCTTATGAGAGTTG | For sequencing BCTV components |
| NbFbox-F | GGCACTCACAAACGTCTATTTC | For qPCR; reference gene |
| NbFbox-R | ACCTGGGAGGCATCCTGCTTAT | For qPCR; reference gene |
| LowBCTV-qRT | CTACACGAAGATGGGCAACCT | For qPCR; amplifies BCTV |
| UpBCTV-qRT | TGACGTCGGAGCTGGATTTAG | For qPCR; amplifies BCTV |
| Rep 2Stop F | GAAAGAGATCACTAAGGATATGTAAGAAATATGTTTTTTGCTTG | For mutagenesis PCR to generate *ΔREP* clones |
| Rep 2Stop R | CATATCCTCAGTGTTCTCTTTCAAAAGAAGAAGCTCTTG | For mutagenesis PCR to generate *ΔREP* clones |
| BCTV C4 C1R | GCGCCGTCTCGCTCAAGCGATGGCCCTTATGAGAGTTGTAC | For subcloning the *ΔIR* clones |
| GUSREP B2F | GCGCCGTCTCGCTCGCCATGATGGCTTCCTCCGAACA | For generating *N. benthamiana* *GUS:NPTII* reporter lines |
| GUSREP B5R | GCGCCGTCTCGCTCAAAGCTCAGAAGAACTCGTCAAGAAG | For generating *N. benthamiana* *GUS:NPTII* reporter lines |
| ZF F B2F | CCGCCGTCTCGCTCGCCATGGCTTCCTCCCCTCCAAA | For subcloning ZFN from the pLSLZDR vector |
| ZF R B5R | GCGCCGTCTCGCTCAAAGCTTAAAAGTTTATCTCACCGTTATTAAATT | For subcloning ZFN from the pLSLZDR vector |

**Table S2.** Sequence Table

| **Sequence name** | **Benchling link** | **Addgene #** | **Brief Description** |
| --- | --- | --- | --- |
| *BCTVDVS-RT-pDGB3alpha2* | https://benchling.com/mattneub/f/lib_Gnbpe9gG-bctv-entry-and-expression-clones/seq_7TBjYG51-bctvvs-rt-pdgb3alpha2/edit | 240730 | pDGB3alpha2 containing the BCTV with the virion sense genes deleted (*BCTVDVS*) and the repair template cargo (*RT*) |
| *BCTVDVS-pUPD2* | https://benchling.com/mattneub/f/lib_Gnbpe9gG-bctv-entry-and-expression-clones/seq_UMty7QQe-bctvvs-pupd2/edit | 240718 | pUPD2 entry vector containing the BCTV with IR with the virion sense genes deleted (*BCTVDVS*) as A1-B1 GB part |
| *BCTVDREP-YPET-pDGB3alpha1* | https://benchling.com/mattneub/f/lib_Gnbpe9gG-bctv-entry-and-expression-clones/seq_z09JsXcC-bctvrep-ypet-pdgb3alpha1/edit | 240725 | pDGB3alpha1 containing the BCTV sequence with an inactive *REP* and *YPET* as cargo |
| *BCTVDREP-RT-pDGB3alpha1* | https://benchling.com/mattneub/f/lib_Gnbpe9gG-bctv-entry-and-expression-clones/seq_mZDCpE8b-bctvrep-rt-pdgb3alpha1/edit | 240728 | pDGB3alpha1 containing the BCTV sequence with an inactive *REP* and the repair template as cargo |
| *BCTVDREP-pUPD2* | https://benchling.com/mattneub/f/lib_Gnbpe9gG-bctv-entry-and-expression-clones/seq_57suOspu-bctvrep-pupd2/edit | 240715 | pUPD2 entry vector containing the BCTV with the virion complementary-sense genes where *Rep* is inactivated. This is a C1 GB part |
| *BCTVDIR-pUPD2* | https://benchling.com/mattneub/f/lib_Gnbpe9gG-bctv-entry-and-expression-clones/seq_YEJ1DGfZ-bctvir-pupd2/edit | 240717 | pUPD2 entry vector containing the BCTV with the virion complementary-sense genes where the *IR* sequence was deleted. This is a C1 GB part |
| *BCTV-RT-pDGB3alpha2* | https://benchling.com/mattneub/f/lib_Gnbpe9gG-bctv-entry-and-expression-clones/seq_iOFgRaQD-bctv-rt-pdgb3alpha2/edit | 240724 | pDGB3alpha2 containing the BCTV with the virion sense genes retained and the repair template as cargo |
| *BCTV-VS domesticated-pUPD2* | https://benchling.com/mattneub/f/lib_Gnbpe9gG-bctv-entry-and-expression-clones/seq_4yL5zWxq-bctv-vs-domesticated-pupd2/edit | 240748 | pUPD2 entry vector containing the BCTV with the virion sense genes domesticated (BsaI site removed) and truncated CP-encoding *V1* |
| *BCTV-YPET-pDGB3alpha2* | https://benchling.com/mattneub/f/lib_Gnbpe9gG-bctv-entry-and-expression-clones/seq_f6R0jbyZ-bctv-ypet-pdgb3alpha2/edit | 240722 | pDGB3alpha2 containing the BCTV sequences and the cargo *YPET* inserted downstream of the truncated CP-encoding *V1* |
| *ZFN-pUPD2* | https://benchling.com/mattneub/f/lib_Gnbpe9gG-bctv-entry-and-expression-clones/seq_6TIfZT95-zfn-pupd2/edit | 240721 | pUPD2 entry vector containing the zinc finger nuclease as a B2-B5 GB part |
| *RT B6-pUPD2* | https://benchling.com/mattneub/f/lib_Gnbpe9gG-bctv-entry-and-expression-clones/seq_TzCGA6XZ-rt-b6-pupd2/edit | 240720 | pUPD2 entry vector containing the repair template as a B6 GB part |
| *BCTVDVSDREP-RT-pDGB3alpha1* | https://benchling.com/mattneub/f/lib_Gnbpe9gG-bctv-entry-and-expression-clones/seq_7bFffndN-bctvvsrep-rt-pdgb3alpha1/edit | 240727 | pDGB3alpha1 containing virion-sense genes deleted (*ΔVS*) and an inactivated Rep gene (*ΔREP*); carries a repair template (*RT*) as its cargo |
| *RT B2-B6-pUPD2* | https://benchling.com/mattneub/f/lib_Gnbpe9gG-bctv-entry-and-expression-clones/seq_mqpcGELD-rt-b2-b6-pupd2/edit | 240719 | pUPD2 entry vector containing the repair template as a B2-B6 GB part |
| *BCTVDVSDREP-YPET-pDGB3alpha1* | https://benchling.com/mattneub/f/lib_Gnbpe9gG-bctv-entry-and-expression-clones/seq_ZS7a0Q9J-bctvvsrep-ypet-pdgb3alpha1/edit | 240726 | pDGB3alpha1 containing BCTV with its virion-sense genes deleted (*ΔVS*) and an inactivated *Rep* gene (*ΔREP*); carries a *YPET* reporter gene as its cargo |
| *35S-ZFN- pDGB3alpha1* | https://benchling.com/mattneub/f/lib_Gnbpe9gG-bctv-entry-and-expression-clones/seq_ieyxsLqD-35s-zfn-pdgb3alpha1/edit | 240729 | pDGB3alpha1 containing the *Zinc Finger Nuclease (ZFN)*, with its expression driven by the constitutive *35S* promoter |
| *BCTV-VS WT-pUPD2* | https://benchling.com/mattneub/f/lib_Gnbpe9gG-bctv-entry-and-expression-clones/seq_5tJwGOyT-bctv-vs-wt-pupd2/edit | 240716 | pUPD2 containing the virion-sense portion of the BCTV genome as a A1-B1 GB part |
| *BeYDV-RT-pUPD2* | https://benchling.com/mattneub/f/lib_Gnbpe9gG-bctv-entry-and-expression-clones/seq_XS4jx3ip-beydv-rt-pupd2/edit | 240747 | pUPD2 containing a BeYDV replicon carrying a GUS:NPTII repair template. Replicon was derived from the pLSLZDR vector (Baltes et al., 2014). |
| *BCTVDVS-YPET-pDGB3alpha1* | https://benchling.com/mattneub/f/lib_Gnbpe9gG-bctv-entry-and-expression-clones/seq_gj9uqlt8-bctvvs-ypet-pdgb3alpha1/edit | 240723 | pDGB3alpha1 containing the BCTV genome with its virion-sense genes deleted (*ΔVS*) and a *YPET* reporter gene as cargo |
| *BCTV-CS-pUPD2* | https://benchling.com/mattneub/f/lib_Gnbpe9gG-bctv-entry-and-expression-clones/seq_SWIywJOA-bctv-cs-pupd2/edit | 240746 | pUPD2 containing the complementary-sense (*CS*) BCTV sequences as a C1 GB part |
