# Supplementary material for: Development of Modular Geminivirus‐Based Vectors for High Cargo Expression and Gene Targeting in Plants
Source: Plant Biotechnol J. 2025 Sep 21;24(2):717–30. doi: 10.1111/pbi.70320 (PMC12906812; doi:10.1111/pbi.70320)
Supplement: Supplementary file 2 — Figure S1: pbi70320‐sup‐0002‐FiguresS1‐S3.pdf. Figure S2: pbi70320‐sup‐0002‐FiguresS1‐S3.pdf. Figure S3: pbi70320‐sup‐0002‐FiguresS1‐S3.pdf. [file PBI-24-717-s001.pdf]

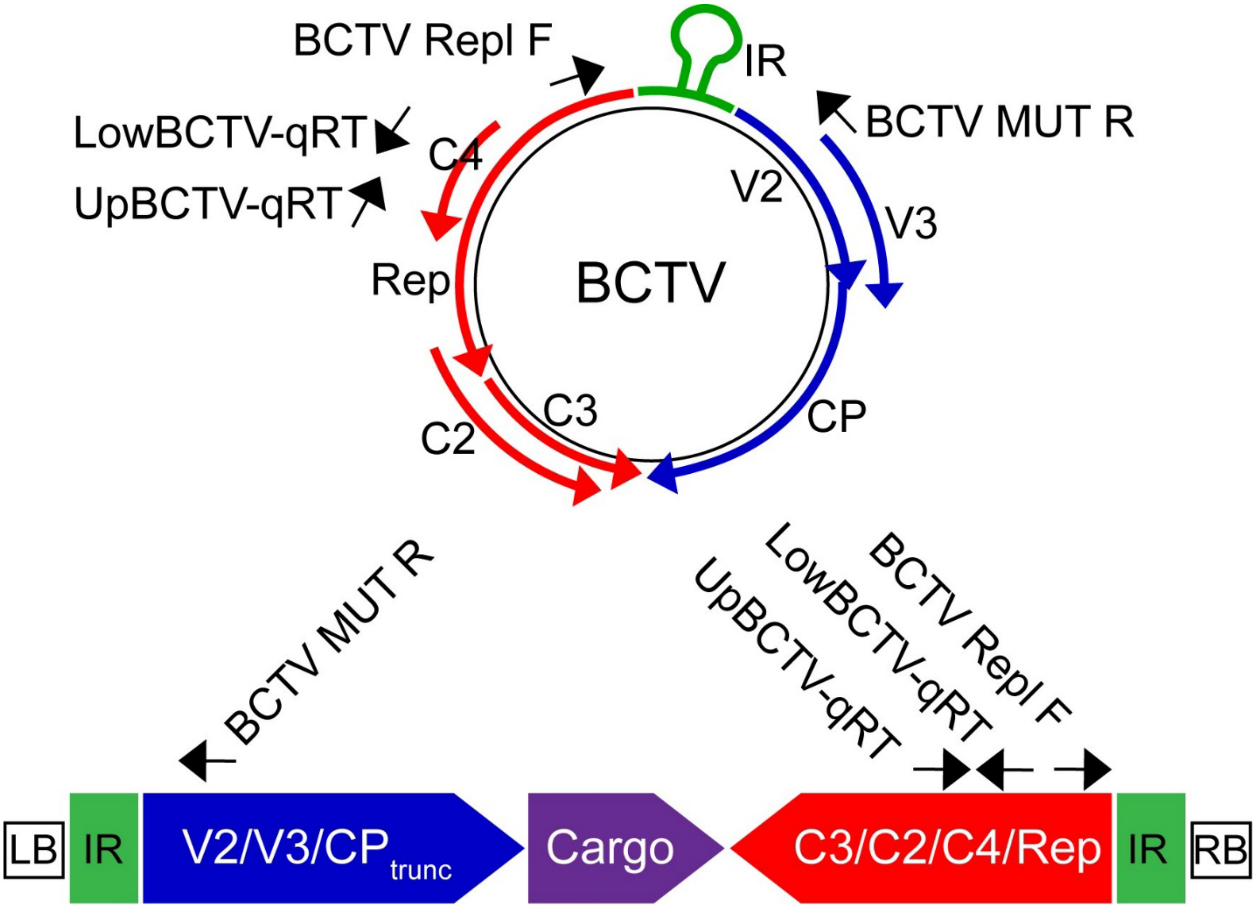

Supporting Figure 1. Diagram depicting the location of primers used in the study to detect the circularized BCTV replicon (BCTV Repl F and BCTV MUT R) and perform qPCR (UpBCTV-qRT and LowBCTV-qRT). BCTV Repl F and BCTV MUT R can only amplify the circular replicon, while UpBCTV-qRT and LowBCTV-qRT can amplify both the circular replicon and non-circularized viral vector.

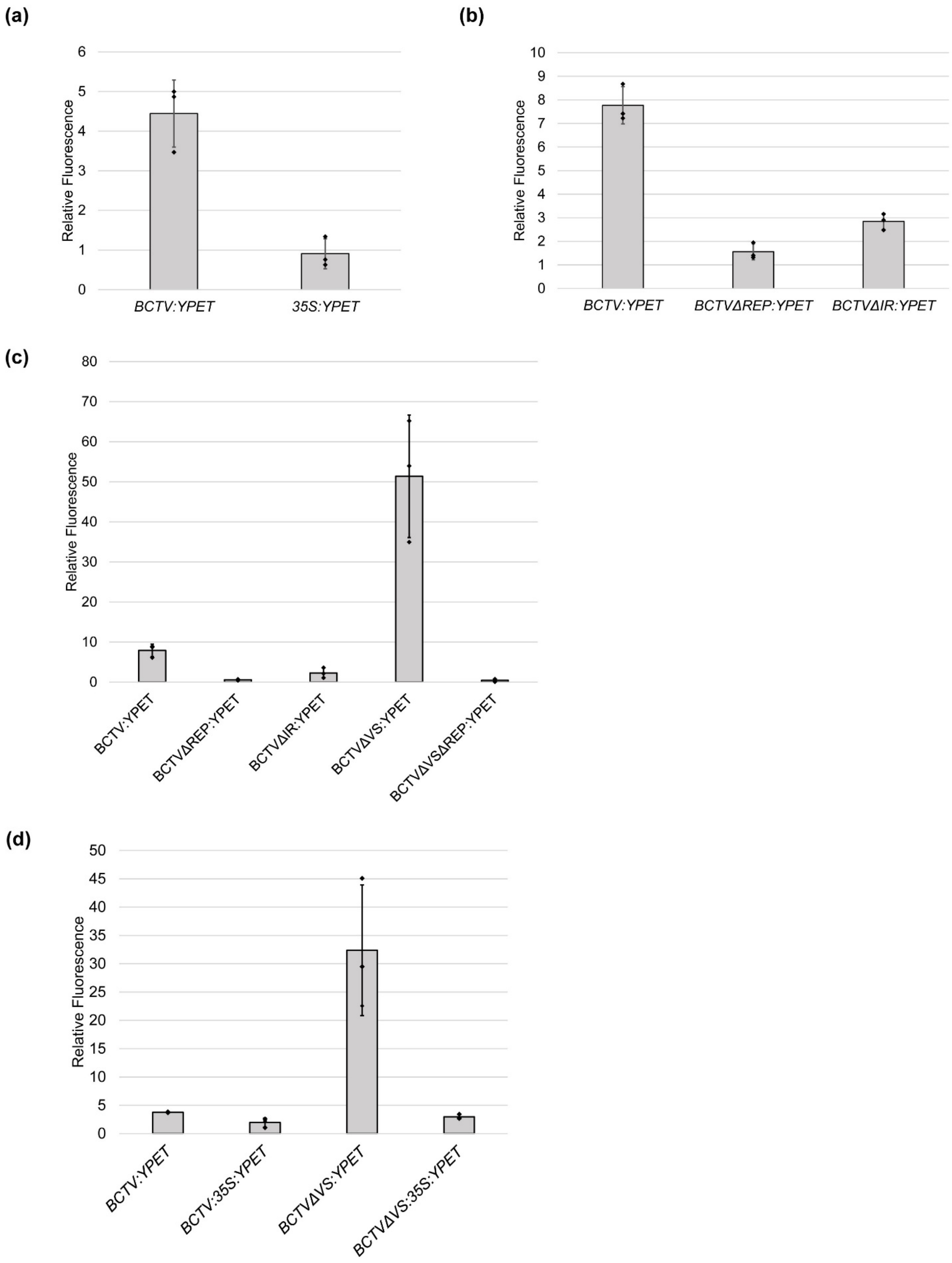
(a)
Relative Fluorescence
0
1
2
3
4
5
6
BCTV:YPET
35S:YPET
(b)
Relative Fluorescence
0
1
2
3
4
5
6
7
8
9
10
BCTV:YPET
BCTVΔREP:YPET
BCTVΔIR:YPET
(c)
Relative Fluorescence
0
10
20
30
40
50
60
70
80
BCTV:YPET
BCTVΔREP:YPET
BCTVΔIR:YPET
BCTVΔVS:YPET
BCTVΔVSΔREP:YPET
(d)
Relative Fluorescence
0
5
10
15
20
25
30
35
40
45
50
BCTV:YPET
BCTV:35S:YPET
BCTVΔVS:YPET
BCTVΔVS:35S:YPET

Supporting Figure 2. Quantification of fluorescence mediated by BCTV vectors harboring *YPET*. Image analysis was performed to compare fluorescence generated by various BCTV vectors. Averages were calculated based on three equal-size areas per each infiltration. Diamonds represent qPCR measurements from individual biological replicates. Analysis was performed using ImageJ.

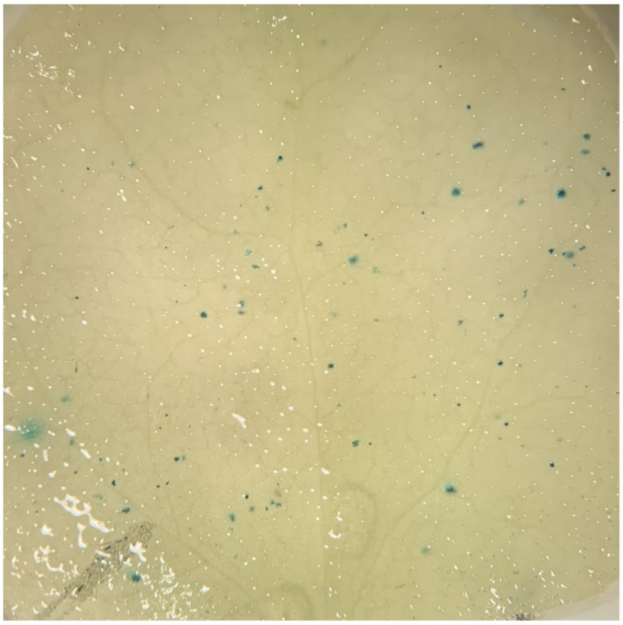

T1 Line 1 + *ZF+BCTV:RT*

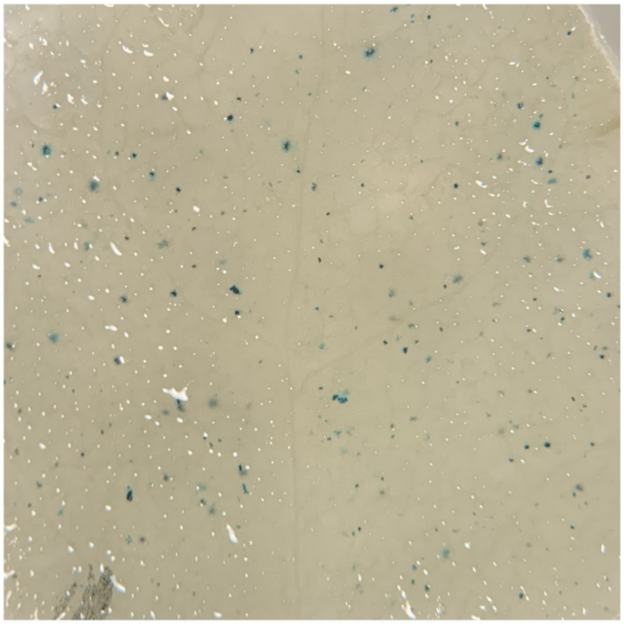

T1 Line 13 + *ZF+BCTV:RT*

Supporting Figure 3. Two functional ‘broken’ *GUS* reporter lines were generated in *N. benthamiana*. Above, T1 plants from lines 1 and 13 were agroinfiltrated with strains carrying BCTV vectors with a *ZFN* that targets the broken reporter and an RT carrying the missing *GUS* sequence. Both lines consistently demonstrated successful repair of the *GUS* reporter and were used in subsequent experiments.
